# CD24 induces the invasion of cholangiocarcinoma cells by upregulating CXCR4 and increasing the phosphorylation of ERK1/2

**DOI:** 10.3892/ol.2013.1587

**Published:** 2013-09-17

**Authors:** KAWIN LEELAWAT, SIRIPORN KEERATICHAMROEN, SURANG LEELAWAT, RUTAIWAN TOHTONG

**Affiliations:** 1 Department of Surgery, Rajavithi Hospital, Rajathevi, Bangkok 10400, Thailand; 2 College of Medicine, Rangsit University, Bangkok 10400, Thailand; 3 Department of Molecular Medicine, Faculty of Science, Mahidol University, Bangkok 10400, Thailand; 4 Biochemistry Laboratory, Chulabhorn Research Institute, Bangkok 10210, Thailand; 5 Department of Phamacognosy, Faculty of Pharmacy, Rangsit University, Pathumthani 12000, Thailand; 6 Department of Biochemistry, Mahidol University, Bangkok 10400, Thailand

**Keywords:** CD24, cholangiocarcinoma, CXC chemokine receptor type 4, extracellular-signal-regulated kinase 1/2, invasiveness

## Abstract

Cholangiocarcinoma is a malignant biliary tract tumor with an extremely poor prognosis. CD24 expression has been linked to the aggressiveness of cholangiocarcinoma cells and the adverse prognosis of cholangiocarcinoma patients. In the present study, the underlying mechanism of aggressive CD24^+^ cholangiocarcinoma cell behavior was elucidated. The magnetic-activated cell sorting system was used to isolate CD24^+^ and CD24^−^ cell populations from RMCCA1 cholangiocarcinoma cells. Using a human tumor metastasis PCR array, it was observed that numerous tumor-associated genes were upregulated in the CD24^+^ cells, including CXC chemokine receptor type 4 (CXCR4). In addition, an intracellular signaling array demonstrated the activation of extracellular signal-regulated kinase (ERK)1/2, which is downstream of the CXCR4 signaling cascade, in the CD24^+^ cells. Inhibition of CXCR4 or ERK1/2 significantly inhibited the motility and invasiveness of the CD24^+^ cells. The present study indicates that CXCR4 and ERK1/2 are induced by CD24 and that these proteins are associated with cholangiocarcinoma cell invasion.

## Introduction

Cholangiocarcinoma arises from the bile duct epithelium and is one of the most common causes of cancer mortality in Thailand (1). Cholangiocarcinoma is one of the most aggressive malignant tumors, being associated with local invasiveness and a high rate of metastasis. The curative treatment of choice is surgery with R0 resection, and there is no effective chemotherapeutic treatment for this type of cancer (2). Therefore, an understanding of the underlying mechanisms, combined with the identification of new molecular markers involved in the regulation of metastasis, is essential for the development of novel therapeutics to treat cholangiocarcinoma patients and improve their survival rates.

CD24 is a heavily glycosylated phosphatidylinositol-anchored mucin-like cell surface protein (3), and it has been detected in activated endothelial cells and platelets (4). Previous studies have demonstrated that CD24 is expressed in a number of types of human malignancies, including lung cancer (5), glioma (6), pancreatic cancer (7), prostatic cancer (8), renal cell carcinoma (9) and ovarian cancer (10). Studies have suggested that CD24 expression may enhance the metastatic potential of tumor cells (11,12). We previously investigated the expression of CD24 in cholangiocarcinoma specimens and its prognostic significance, and our results demonstrated that high CD24 expression was significantly correlated with lymph node metastasis and positive surgical margins in cholangiocarcinoma patients (12). In addition, we demonstrated that CD24 expression was significantly associated with the overall survival of these patients. For *in vitro* studies, we isolated CD24^+^ and CD24^−^ cell populations from RMCCA1 cholangiocarcinoma cells using the magnetic-activated cell sorting (MACS) system. The results showed that CD24^+^ RMCCA1 cells had significantly increased migration and invasion capabilities compared with CD24^−^ cells (12). However, the underlying mechanisms of the CD24 induction of cholangiocarcinoma cell migration and invasion have yet to be investigated. Therefore, the purpose of the present study was to investigate the roles of CD24 in the migration and invasion of cholangiocarcinoma and to identify the targets induced by CD24 in cholangiocarcinoma cells.

In the present study, the differential expression of genes associated with cancer metastasis in CD24^+^ and CD24^−^ cells was investigated using the Human Tumor Metastasis RT^2^ Profiler™ PCR array (Qiagen, Valencia, CA, USA). In addition, the phosphorylation of signaling molecules mediated by CD24 expression was examined using the PathScan^®^ Intracellular Signaling array kit (Cell Signaling Technology, Beverly, MA, USA).

## Materials and methods

### Cell culture

The human cholangiocarcinoma cancer cell line RMCCA1, which we previously derived from a peripheral cholangiocarcinoma patient (13), was used in the present study. The study was approved by the ethics committee of Rajavithi Hospital (Bangkok, Thailand). The cells were grown in Ham’s F-12 medium (Invitrogen Life Technologies, Carlsbad, CA, USA) supplemented with 10% fetal bovine serum (FBS) and 1% penicillin/streptomycin. In all experiments, the cells were maintained at 37°C in a humidified 5% CO_2_ incubator.

### Isolation of CD24^+^ and CD24^−^ populations by magnetic cell sorting

RMCCA1 cells were incubated with a fluorescein isothiocyanate (FITC*)*-conjugated anti-human CD24 antibody (Miltenyi Biotec, Auburn, CA, USA), magnetically labeled with anti-FITC microbeads and separated on a MACS MS column (Miltenyi Biotec), according to the manufacturer’s instructions. The magnetic separation step was repeated twice to obtain highly purified cell populations.

### Quantitative (q)PCR

The cells were harvested by trypsinization and total RNA was extracted using the RNeasy mini kit (Qiagen), according to the manufacturer’s protocol. Gene expression was quantified by qPCR using the SuperScript^®^ III Platinum^®^ SYBR^®^-Green One-Step qRT-PCR kit (Invitrogen Life Technologies). Thermal cycling was performed using the following steps: 50°C for 3 min, followed by 95°C for 5 min, 40 cycles of 95°C for 15 sec and 60°C for 30 sec. The primer sequences used to amplify the genes are shown in Table I. The relative quantitation of gene expression against an internal control (GAPDH) was calculated using the comparative Ct method.

### Human tumor metastasis PCR-array

RNA (1 μg) was reverse transcribed using an RT^2^ First Strand kit (Qiagen) and applied to the Human Tumor Metastasis RT^2^ Profiler PCR array (PAHS-028A-2; Qiagen), which was used to analyze the expression of 84 genes involved in metastasis. The reaction conditions were as follows: 95°C for 10 min, followed by 40 cycles at 95°C for 15 sec, 55°C for 40 sec and 72°C for 30 sec. For data analysis, the RT^2^ Profiler PCR array software package was used. Genes exhibiting a minimum 3-fold increase/decrease or genes with significantly different normalized cycle threshold values were considered to have altered expression.

### Cell viability assay

The cells were seeded into 96-well culture plates at a density of 10,000 cells/well, followed by the addition of vehicle (normal medium) or various concentrations of AMD3100 (0.1–10 μg/ml) or U0126 (0.5–10 μM). The cells were then incubated for 24 h. The number of viable cells after the drug treatment was assessed using the water-soluble tetrazolium salt (WST)-1 Cell Proliferation Assay Reagent (Roche Diagnostics, Laval, QC, Canada) according to the recommendations of the manufacturer. The degree of cell proliferation was assessed by determining the *A*_450_ nm of the cell culture medium after the addition of WST-1 for 2 h.

### Cell motility (wound-healing) assay

Wounds were generated by puncturing confluent cultures of cells with 20-μl pipette tips. These wounds were then covered with Matrigel (Becton-Dickinson Bioscience, Bedford, MA, USA) to mimic cell communication with the microenvironment. The areas of wounds were marked and images were captured. The distance of cell movement toward the wound space was assessed following 24 h.

### In vitro invasion assay

The invasiveness of the cholangiocarcinoma cells was assayed in a 24-well BioCoat Matrigel invasion chamber (8-μm; Becton-Dickinson Bioscience). The upper chamber was seeded with 50,000 cells and the lower chamber contained 1% FBS. Subsequent to 24 h of incubation, the invading cells on the lower surface of the Matrigel-coated membrane were fixed with 25% methanol, stained with crystal violet (Sigma Chemical Co., St. Louis, MO, USA) and counted in 5 random ×400-power fields under a light microscope.

### PathScan sandwich immunoassay

The PathScan Intracellular Signaling array kit (Cell Signaling Technology) was used, according to the manufacturer’s instructions, to simultaneously detect 18 significant and well-characterized signaling molecules that were phosphorylated or cleaved. Briefly, the cells were washed with ice-cold 1X phosphate-buffered saline and lysed in 1X Cell Lysis buffer. The Array Blocking Buffer was added to each well and incubated for 15 min at room temperature. Subsequently, the lysate was added to each well and incubated for 2 h at room temperature. Subsequent to washing, the detection antibody cocktail was added to each well and incubated for 1 h at room temperature. Horseradish peroxidase (HRP)-linked streptavidin was added to each well and incubated for 30 min at room temperature. The slide was then covered with LumiGLO/Peroxide reagent (Cell Signaling Technology) and exposed to film for 2–30 sec.

### Statistical analysis

The experiments were performed in triplicate and each result was reported as the mean±standard deviation. Data were compared using a paired, two-tailed Student’s t-test. P<0.05 was considered to indicate a statistically significant difference.

## Results

### CD24^+^ cells exhibit upregulated expression of multiple metastatic genes

We previously reported that high CD24 expression significantly correlated with the poor clinical outcomes of cholangiocarcinoma patients and the increased invasiveness of cholangiocarcinoma cells *in vitro*(12). However, little is known about the actual mechanisms by which CD24 mediates these effects. To investigate the differences between the molecular mechanisms of metastasis in CD24^+^ and CD24^−^ cells, total RNA from CD24^+^ and CD24^−^ cells was extracted and analyzed by qPCR using the Human Tumor Metastasis RT^2^ Profiler PCR array. The relative expression levels of 84 genes involved in metastasis, normalized to 4 housekeeping genes (B2M, HPRT1, RPL13A and GAPDH), were determined in a 96-well plate format. Using a threshold value of 3-fold expression change, it was observed that CD24 expression affected the expression of multiple metastatic genes. The results showing the up- and downregulated genes in the CD24^+^ versus CD24^−^ cells are presented in Table II. The CD24^+^ cells exhibited elevated expression levels of 27 genes, consisting of several matrix metalloproteinases (MMP2, MMP9 and MMP13), MMP inhibitors (TIMP3 and TIMP4), cell adhesion genes (APC, CDH11, ITGA7, SYK and FN1), cell growth and proliferation genes (GNRH1, SSTR2, TSHR, HGF, IGF1, CCL7, CXCL12, TNFSF10, CXCR4, FGFR4, KISS1R, TRPM1 and IL8RB) and other genes associated with metastasis (CST7, CTSK, CTSL1 and ACTB).

**Figure 1:**
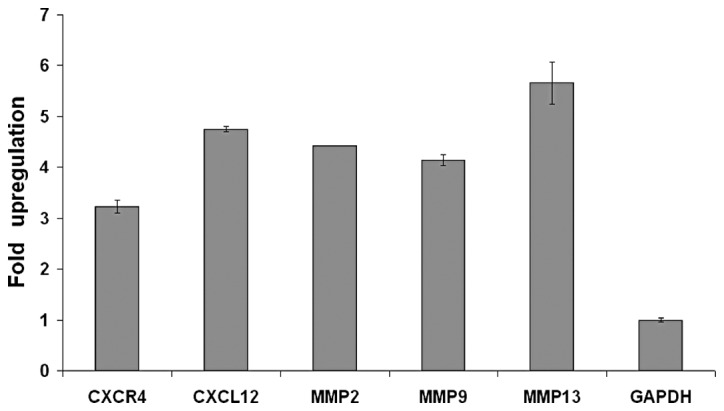
Validation of expression changes for 5 upregulated genes in CD24^+^ cells by quantitative (q)PCR analysis. Gene expression levels from the Human Tumor Metastasis RT^2^ Profiler™ PCR array were validated for 5 genes that are markedly correlated with tumor metastasis. Data are shown as the fold upregulation in CD24^+^ over CD24^−^ cells. CXCR4, CXC chemokine receptor type 4; MMP, matrix metalloproteinase.

The 5 randomly selected gene expression changes observed using the Human Tumor Metastasis RT^2^ Profiler PCR array (CXCR4, CXCL12, MMP2, MMP9 and MMP13) were confirmed using qPCR. The increased expression levels of all 5 genes were confirmed in the CD24^+^ cells using a threshold value of 3-fold expression change (Fig. 1).

### Role of CXCR4 in CD24^+^ and CD24^−^ cell motility and invasion

**Figure 2:**
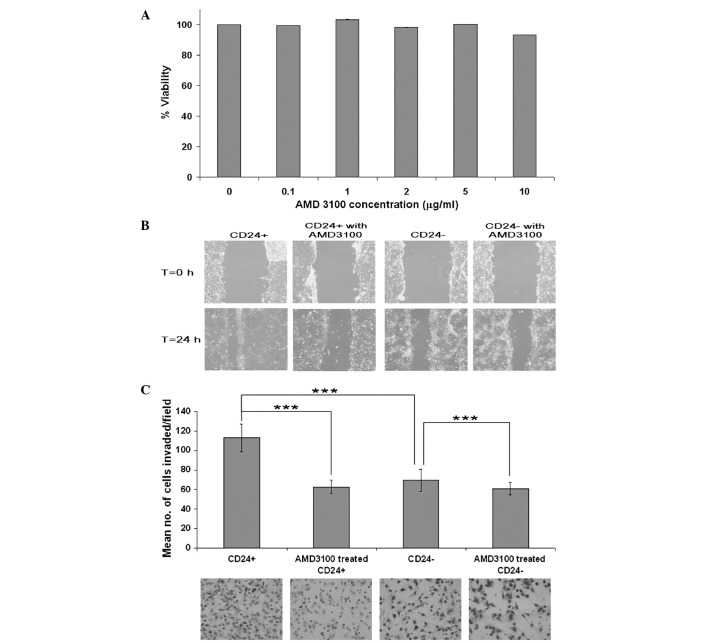
(A) Cell viability assay. The cells were treated with various concentrations of AMD3100 (0, 0.1, 1, 2, 5 and 10 μg/ml) for 24 h. The number of viable cells following the treatment was assessed using a water-soluble tetrazolium salt (WST)-1 cell proliferation assay. (B) CD24^+^ and CD24^−^ cell motility towards Matrigel (treated and untreated with AMD3100). Wounds were made in confluent cells and the cells were allowed to heal for 24 h. (C) CD24^+^ and CD24^−^ cell invasion capabilities (treated and untreated with AMD3100). Transwells were pre-coated with Matrigel and the cells were allowed to invade for 20 h and then counted. Data are presented as the mean ± SD. ^***^P<0.001, significant difference in the cell number.

We previously demonstrated that the chemokine receptor CXCR4 had a major role in the invasiveness of cholangiocarcinoma cells. To study the correlation between CXCR4 expression and the motility and invasion of CD24^+^ and CD24^−^ cells, CXCR4 activity was inhibited using AMD3100, a non-competitive antagonist of CXCR4. First, the effects of AMD3100 on cell viability were tested; the cells were treated with various concentrations of AMD3100 (0, 0.1, 1, 2, 5 and 10 μg/ml) for 24 h. The WST-1 cell proliferation assay was used to detect the number of viable cells following treatment. The results revealed that blocking CXCR4 activity with 0–10 μg/ml AMD3100 did not significantly affect cell viability (Fig. 2A). Cell motility and invasion experiments were then performed using AMD3100 at a concentration of 5 μg/ml.

To evaluate the effect of AMD3100 on cell motility, a wound-healing assay was performed. The results showed that administering AMD3100 clearly suppressed the cell motility of CD24^+^ cells towards Matrigel. By contrast, CD24^−^ cells were largely unaffected (Fig. 2B). To investigate whether CXCR4 modulation affected the invasive ability of the cells, a cell invasion assay was performed. In the presence of AMD3100, the invasion ability was significantly inhibited in the CD24^+^ cells (the number of invasive cells was 55% compared with the untreated cells) and CD24^−^ cells, although to a lesser extent (the number of invasive cells was 87% compared with the untreated cells; Fig. 2C).

### Role of ERK1/2 in CD24^+^ and CD24^−^ cell motility and invasion

**Figure 3:**
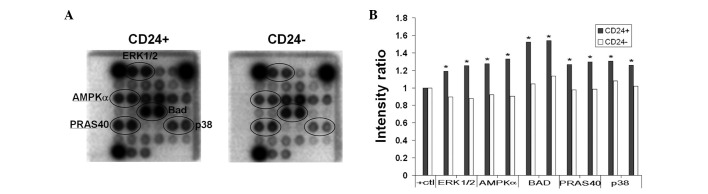
(A) Chemiluminescent array images of the PathScan Intracellular Signaling array kit revealing various phosphorylated signaling nodes. CD24^+^ cells stimulate the phosphorylation of extracellular-signal-regulated kinase (ERK)1/2 at Thr202/Tyr204, AMP-activated protein kinase α (AMPKα) at Thr172, PRAS40 at Thr246, Bad at Ser112 and p38 at Thr180/Tyr182. Images were captured following brief exposure of the slide to standard chemiluminescent film. (B) Array image pixel intensity ratio of phosphorylated signaling molecules. ^*^P<0.05, vs. control.

To elucidate the signal mediation of the CD24^+^ cells, the phosphorylation of 18 significant and well-characterized signaling molecules was simultaneously examined using the PathScan Intracellular Signaling array kit. The results showed that the phosphorylation of multiple signaling molecules, including ERK1/2, AMP-activated protein kinase α (AMPKα), PRAS40, Bad and p38, was higher in the CD24^+^ cells compared with the CD24^−^ cells (Fig. 3).

**Figure 4:**
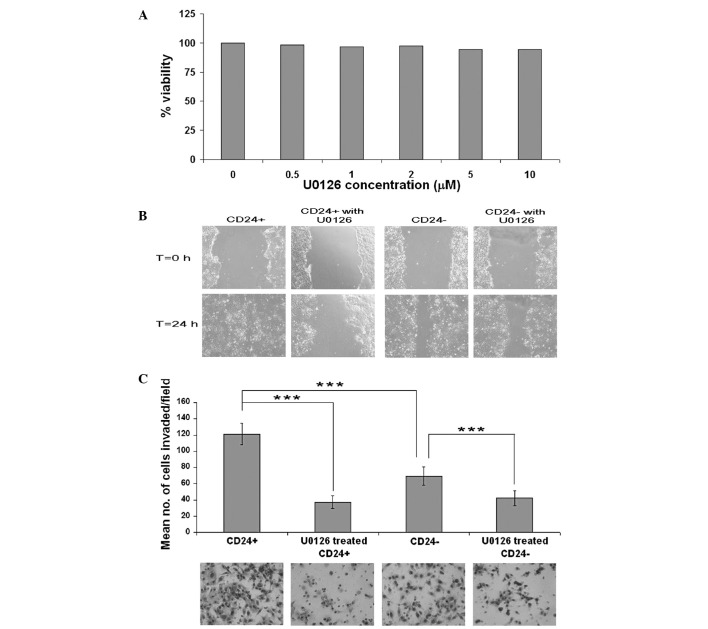
(A) Cell viability assay. The cells were treated with various concentrations of U0126 (0, 0.5, 1, 2, 5 and 10 μg/ml) for 24 h. The number of viable cells following the treatment was assessed using a water-soluble tetrazolium salt (WST)-1 cell proliferation assay. (B) CD24^+^ and CD24^−^ cell motility towards Matrigel (treated and untreated with U0126). Wounds were made in confluent cells and the cells were allowed to heal for 24 h. (C) CD24^+^ and CD24^−^ cell invasion capabilities (treated and untreated with AMD3100). Transwells were pre-coated with Matrigel and the cells were allowed to invade for 20 h and then counted. Data are presented as the mean ± SD. ^***^P<0.001, significant difference in the cell number.

Previous studies on CXCR4/CXCL12 have reported that CXCL12 induces proliferation and survival through ERK1/2 activation (14,15). These results are supported by our previous data, which showed that the addition of a CXCR4 inhibitor (AMD3100) abrogated the CXCL12-induced phosphorylation of mitogen-activated protein kinase (MEK)1/2 in cholangiocarcinoma cells (16). Therefore, the role of ERK1/2 in CD24-mediated cell motility and invasion in the CD24^+^ cells was investigated. The cells were treated with U0126 (a MEK/ERK inhibitor) and tested using a wound-healing assay and a cell invasion assay. U0126 was used at a concentration of 2 μM, which had no significant effect on cell viability compared with the untreated cells based on the results of the cell viability assay (Fig. 4A). The results of the wound-healing assay showed that U0126 significantly inhibited the motility of the CD24^+^ cells, while a more muted effect was detected in the CD24^−^ cells (Fig. 4B). Additionally, the invasion assay showed that U0126 treatment significantly decreased the numbers of invading CD24^+^ and CD24^−^ cells, although the treatment appeared to have a more pronounced effect on the CD24^+^ cells (Fig. 4C).

## Discussion

An expanding body of literature supports the role of CD24 in controlling gene expression (17–20). The observed effects of CD24 on cell invasion indicate that its expression may affect tumor invasiveness by altering the expression of genes involved in metastasis. In the present study, the PCR array system was used to focus on human tumor metastasis and identify genes associated with metastasis that are affected by CD24 expression. The genes selected for this array encoded several classes of protein factors, including those involved in the cell cycle and cell adhesion, growth, proliferation and apoptosis, plus similar extracellular matrix (ECM) components, transcription factors and regulators and other genes associated with tumor metastasis. Using qPCR, the expression of a focused panel of genes associated with metastasis was analyzed. It was observed that the expression levels of 27 genes were significantly increased in the CD24^+^ cells. Of these overexpressed genes, 5 (CXCR4, CXCL12, MMP2, MMP9 and MMP13) were selected on the basis of their strong correlation with tumor metastasis and were validated by qPCR. As expected, all 5 genes were demonstrated to be upregulated by at least 3-fold in the CD24^+^ cells. These results indicate that CD24 is a potential potent regulator of gene expression.

To the best of our knowledge, the present study is the first to demonstrate the association between CD24 and CXCR4 in cholangiocarcinoma. The CXCL12/CXCR4 system is an important mediator of invasion and metastasis. Researchers have observed that the CXCR4-mediated activation of signaling pathways results in increased cell migration and invasion associated with actin polymerization and MMP-9 activation (21). The present results are consistent with those of previous studies that demonstrated that CXCR4 inhibition with the non-peptide small molecule AMD3100 significantly inhibits cancer cell invasion (22,23). The present study demonstrated that blocking CXCR4 signaling with AMD3100 results in decreased motility and invasion ability in CD24^+^ and CD24^−^ cells (although to a greater extent in CD24^+^ cells), indicating that CXCR4 is important in cholangiocarcinoma cell invasiveness.

Results have been published that suggest the involvement of CD24 in intracellular signaling (24–27). Experiments using B-lymphocytes have shown that incubation with a monoclonal antibody against CD24 triggered an increase in free cytoplasmic calcium (24) and a physical interaction of CD24 with members of the Src kinase family (25–27), demonstrating the signal-transducing capabilities of CD24. The aim of the present study was to assess the signal transduction potential of CD24 by identifying the putative signaling molecules associated with CD24 in cholangiocarcinoma. The results from the PathScan Intracellular Signaling array kit demonstrated that the CD24^+^ cells exhibit higher levels of ERK1/2, AMPKα, PRAS40, Bad and p38 phosphorylation compared with the CD24^−^ cells. These findings are consistent with those of Wang *et al*(28), showing that CD24-dependent ERK1/2 and p38 MAPK activation are required for colorectal cancer cell proliferation *in vitro* and *in vivo*. The targeting of CD24 by a specific siRNA consistently results in the reduced phosphorylation of Lyn, ERK1/2 and p38 MAPK in SW480^CD24^(29).

As previous studies have demonstrated that ERK1/2 phosphorylation occurs through CXCR4 activation, the present study focused on ERK1/2, which is significant in regulating the malignant potential of cancer cells (30). The contribution of ERK1/2 to CD24-induced motility and invasion was examined using a specific inhibitor of the upstream ERK1/2 activator MEK1/2 (U0126). Consistent with previous studies (16,31), the present data showed that U0126 abrogates CD24-induced wound-healing and invasion in the CD24^+^ and CD24^−^ cells (although this inhibition was less apparent in the CD24^−^ cells), indicating that ERK1/2 activation is markedly correlated with CD24 expression in cholangiocarcinoma. The present study demonstrated that CD24 is associated with the upregulation of CXCR4. The present results and those of our previous studies show that ERK1/2 is a downstream component of CD24 and CXCR4 signaling. Thus, we hypothesize that the activation of the MAPK/ERK pathway may be the potential mechanism of CD24-mediated cell invasiveness and that the difference in responsiveness to AMD3100 and U0126 between the CD24^+^ and CD24^−^ cells indicates that distinct invasive signaling pathways may operate in these two cell lines.

Furthermore, using the PCR and intracellular signaling array systems, it was observed that numerous tumor metastasis-associated genes and intracellular signaling molecules in the CD24^+^ cells are upregulated. Additional studies are required to determine whether these molecules affect the aggressiveness of CD24^+^ cholangiocarcinoma cells.

In conclusion, the results of the present study showed that CD24 has a major role in cholangiocarcinoma cell invasion. This effect is associated with the upregulation of several factors, particularly CXCR4, and the phosphorylation of ERK1/2. These findings indicate that CD24 agonists should be studied as novel drugs for the treatment of cholangiocarcinoma.

## Figures and Tables

**Table I:** Primers used for qPCR analysis.

Gene name	Forward sequence 5′→3′	Reverse sequence 5′→3′
CXCR4	ACTTCAGTTTGTTGGCTGCGGC	ACCGCTGGTTCTCCAGATGCG
CXCL12	CGTGGGGGAGGGGGCCTTAAC	CAACGTGCACAGGTACAGGGCA
MMP2	GATGGCACCCATTTACACCTAC	GTCCTTGAAGAAGAAGATCTC
MMP9	CCTTCTACGGCCACTACT	GAGAATCGCCAGTACTTCCCAT
MMP13	CTTAGAGGTGACTGGCAAAC	GCCCATCAAATGGGTAGAAG
GAPDH	TGCACCACCAACTGCTTAGC	GGCATGGACTGTGGTCATGAG

GAPDH gene expression was used as an internal control. CXCR4, CXC chemokine receptor type 4; MMP, matrix metalloproteinase; qPCR, quantitative PCR.

**Table II:** Fold difference of gene expression between the CD24^+^ and CD24^−^ cells.

Gene	Fold up- or downregulation
ACTB	**34.84**
TIMP4	**26.04**
KISS1R	**16.59**
CXCL12	**12.84**
APC	**12.66**
FGFR4	**10.80**
TNFSF10	**10.57**
CXCR4	**8.83**
TSHR	**8.24**
IGF1	**7.74**
MMP13	**7.37**
IL8RB	**7.07**
TIMP3	**6.98**
MMP2	**6.46**
SSTR2	**6.29**
CCL7	**6.20**
FN1	**5.95**
HGF	**5.79**
GNRH1	**5.55**
TRPM1	**4.70**
CST7	**4.01**
MMP9	**4.01**
CDH11	**3.79**
SYK	**3.59**
CTSL1	**3.39**
CTSK	**3.28**
ITGA7	**3.25**
MMP3	2.87
COL4A2	2.85
MMP11	2.74
MMP10	2.43
MCAM	2.42
KISS1	2.30
ETV4	2.25
FLT4	2.18
MYCL1	2.15
ITGB3	2.10
NR4A3	2.06
EPHB2	1.90
RORB	1.88
MMP7	1.86
MTSS1	1.73
METAP2	1.71
TP53	1.56
SMAD2	1.47
HPSE	1.44
HPRT1	1.39
CDH1	1.38
IL18	1.33
CHD4	1.32
PNN	1.31
SRC	1.30
PLAUR	1.29
RB1	1.29
PTEN	1.28
MYC	1.25
VEGFA	1.25
MGAT5	1.23
NME2	1.23
FAT1	1.22
DENR	1.22
RPSA	1.22
CDKN2A	1.22
MET	1.20
TIMP2	1.17
MTA1	1.15
IL1B	1.13
EWSR1	1.10
SMAD4	1.10
KRAS	1.08
TGFB1	1.07
TCF20	1.02
CTNNA1	1.00
NME1	0.99
RPL13A	0.99
BRMS1	0.99
SET	0.97
NF2	0.97
MDM2	0.95
NME4	0.95
HRAS	0.95
CTBP1	0.92
GAPDH	0.87
CD82	0.85
HTATIP2	0.85
CD44	0.84
B2M	0.83
FXYD5	0.75

Values in bold denote significant up- or downregulation (fold change >3).
